# Phytochemicals as Multitarget Therapeutics in Pancreatic Cancer: Mechanisms, Clinical Evidence, and Translational Challenges

**DOI:** 10.1002/ptr.70430

**Published:** 2026-08-16

**Authors:** Md. Rezaul Islam, Abdur Rauf, F. M. Shourav, Hanan A. Ogaly, Abdullah S. M. Aljohani, Waleed Al Abdulmonem, Kamala Badalova, Marcello Iriti

**Affiliations:** ^1^ Department of Pharmacy, Faculty of Health and Life Sciences Daffodil International University Dhaka Bangladesh; ^2^ Department of Chemistry University of Swabi Anbar Khyber Pakhtunkhwa Pakistan; ^3^ Department of Cardiothoracic Surgery The First Affiliated Hospital of Chongqing Medical University Chongqing China; ^4^ Chemistry Department College of Science, King Khalid University Abha Saudi Arabia; ^5^ Department of Medical Biosciences College of Veterinary Medicine, Qassim University Buraydah Saudi Arabia; ^6^ Departmentof Pathology College of Medicine, Qassim University Buraydah Saudi Arabia; ^7^ Pharmaceutical Toxicology and Chemistry Department Azerbaijan Medical University Baku Azerbaijan; ^8^ Department of Biomedical, Surgical and Dental Sciences University of Milan Milan Italy; ^9^ National Interuniversity Consortium of Materials Science and Technology (INSTM) Firenze Italy

**Keywords:** clinical insights, multitarget therapy, pancreatic cancer, phytochemicals, signaling pathways

## Abstract

Pancreatic cancer, especially pancreatic ductal adenocarcinoma, is known for its aggressive nature, late diagnosis, and resistance to standard treatments. This review demonstrates the therapeutic potential of phytochemicals as multitarget agents in pancreatic cancer, emphasizing their molecular mechanisms, pharmacological properties, and translational relevance. Important carcinogenic signaling pathways, such as KRAS/MAPK, PI3K/Akt, NF‐κB, STAT3, Hedgehog, and Wnt/β‐catenin, are modulated by phytochemicals like curcumin, quercetin, resveratrol, and others. These substances demonstrate anticancer effects by suppressing epithelial–mesenchymal transition, inducing apoptosis, reducing proliferation and angiogenesis, and modulating oxidative stress. Preclinical research indicates consistent multipathway targeting and potential enhanced efficacy with traditional chemotherapeutics, although evidence from clinical studies remains limited and inconsistent. New strategies to improve drug stability, target tumors more effectively, and enhance treatment outcomes involve the use of combination medicines and nanotechnology‐based delivery systems. The gap between preclinical effectiveness and clinical translation continues due to insufficient large‐scale trials, diverse study designs, and a lack of standardized dosing strategies. Phytochemicals show potential as multitarget treatments for pancreatic cancer, but their clinical application is inhibited by pharmacokinetic challenges and insufficient validation.

## Graphical abstract

Pancreatic cancer, particularly pancreatic ductal adenocarcinoma, is aggressive and often diagnosed late, making treatment challenging. This review highlights the potential of phytochemicals, such as curcumin and quercetin, as multitarget agents that influence key carcinogenic pathways. These compounds exhibit anticancer effects by inhibiting processes such as epithelial‐mesenchymal transition and angiogenesis. Combination therapies and nanotechnology may enhance treatment efficacy.

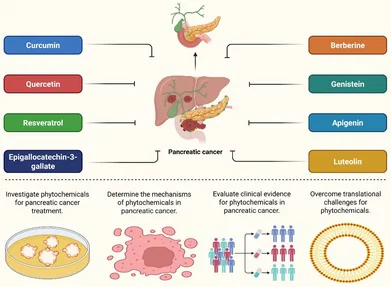

## 1 Introduction

Pancreatic cancer (PC) develops when cancer cells proliferate in the pancreatic tissue. PC is a difficult tumor to treat due to its subtle symptoms, limited treatment options, and severe side effects. Typical symptoms of PC are weight loss, low appetite, back or stomach pain, and jaundice (Oberstein and Olive 2013). In 2018, global cancer statistics showed 432,242 fatalities and 458,918 newly diagnosed cases, representing 2.5% and 4.5% of all cases, respectively. PC ranked as the sixth most prevalent cancer among both men and women (Bray et al. 2018). For most PC patients, surgery is a vital curative treatment. The surgical resection success rate is low, with only 15%–20% of patients eligible, as 80%–85% of cases are diagnosed at an advanced stage (Paulson et al. 2013). Additionally, PC has a low 5‐year survival rate of under 5%, primarily due to defects in apoptotic signaling and mutations in genes like K‐ras and p53. Resistance to apoptosis and invasion is associated with several factors, including NF‐κB activation and overexpression of heat shock proteins. Abnormal activation of multiple pathways (MAPK, PI3K–AKT, JAK/STAT, SHH, Notch, Wnt/β‐catenin) is also associated with this resistance. Targeting pathways involved in apoptosis resistance may be effective using herbal medicines or other natural products, which show potential when used alone or in combination with traditional anticancer drugs such as Gemcitabine (Li and Leung 2014). KRAS, TGF‐β, Notch, HIF, and Wnt/β‐catenin are key signaling pathways that enhance tumor growth and promote resistance (Argentiero et al. 2024). Phytochemicals from natural sources offer a valuable resource for developing new treatments for various diseases. The induction of apoptosis, initiation of cell cycle arrest, inhibition of kinases, and blocking of carcinogens are ways in which phytochemicals exert anticancer effects (Majrashi et al. 2023). Certain herbal medicine compounds may induce apoptosis in PC cells. The anticancer effects of natural herbal products were explored, including associated signaling pathways, such as JAK–STAT, death receptor‐mediated, mitochondrial‐dependent, NF‐κB‐mediated, MAPK‐mediated, ERS‐mediated, and PI3K‐Akt‐mediated apoptotic pathways (Li et al. 2022). Numerous plants contain the flavonoids quercetin and resveratrol, which have been studied for their effects on the epithelial‐mesenchymal transition (EMT) in PC cells, specifically CD133+ and CD133− cells. Quercetin increased TNF‐α and vimentin immunoreactivity and decreased ACTA‐2, IL‐1β, and N‐cadherin immunoreactivity. Resveratrol reduced TNF‐α and N‐cadherin in CD133+ cells (Hoca et al. 2020). Quercetin and luteolin on the growth and EGFR tyrosine kinase activity of MiaPaCa‐2 cancer cells. Exposure to 20 μM of quercetin and luteolin led to reduced cell proliferation and decreased phosphorylation of proteins, including EGFR. Quercetin and luteolin target cells sensitive to neoplastic development by inhibiting PTK activities, which are critical for EGFR autophosphorylation. Additionally, quercetin and luteolin induced apoptosis, marked by DNA fragmentation and PARP degradation. Quercetin and luteolin modulate the EGF‐mediated signaling pathway, significantly inhibiting MiaPaCa‐2 cell growth and promoting apoptosis, with EGFR kinase regulation (Lee et al. 2002). Curcumin shows promise in preventing or treating PC by inhibiting malignant neoplasm growth and reducing cell proliferation (Wang et al. 2008). This review demonstrates phytochemicals as multitarget agents, highlighting their mechanisms, comparing evidence levels, and discussing translational challenges and clinical relevance.

## 2 Methodology

A comprehensive literature search was conducted using electronic databases, including PubMed, Scopus, and Web of Science, to identify relevant studies on phytochemicals in PC. Search terms and Boolean combinations included “pancreatic cancer,” “phytochemicals,” “natural compounds,” “signaling pathways,” and “clinical evidence.” Original research articles and review papers reporting molecular mechanisms, preclinical findings (in vitro and in vivo), and clinical evidence related to phytochemicals in PC were included. Non‐English publications, non‐peer‐reviewed articles, and studies lacking relevance were excluded. Duplicates were removed prior to screening, and relevant studies were selected based on title, abstract, and full‐text evaluation.

## 3 Pathogenesis

Pancreatic ductal adenocarcinoma (PDAC) (Figure 1), originating from ductal epithelial cells, is characterized by its aggressive nature and poor prognosis. The disease exhibits significant genetic and phenotypic variability, complicating the development of effective therapies. Genetic alterations in PDAC include activating mutations in the KRAS oncogene and inactivating mutations in tumor suppressor genes such as TP53, SMAD4, and CDKN2A/p16, contributing to tumor aggressiveness. PDAC can be classified into three to four molecular subgroups based on distinct phenotypic and molecular characteristics (Grant et al. 2016). PDAC is difficult to treat because of late detection and treatment resistance. The disease's pathophysiology, risk factors, molecular changes, and current surveillance practices for high‐risk groups are crucial (Wood et al. 2022). CDK7 inhibition has been tested for treating PDAC progression in various preclinical mouse models. Kinase inhibition leads to decreased gene transcription and suppression of the cell cycle. Inhibiting CDK7‐dependent transcriptional addiction in cancer is a useful strategy for reducing disease aggressiveness (Alrouji et al. 2025; Lu et al. 2019). The molecular pathophysiology of PC is essential for the development of novel preventive and therapeutic strategies. Primary mechanisms include the activation of oncogenes, inactivation of tumor suppressor genes, and the dysregulation of critical signaling pathways, particularly, the EGFR, Akt, and NF‐κB pathways (Sarkar et al. 2007). PC arises from both acquired and inherited gene mutations associated with cancer, notably affecting tumor‐suppressor genes (p16/CDKN2A, TP53, and SMAD4), oncogenes (KRAS, BRAF, AKT2, MYB, and AIB1), and genome‐maintenance genes (MLH1 and MSH2). Genetic alterations facilitate early detection of pancreatic neoplasia, the development of mechanism‐based therapies for advanced cases, and provide a basis for genetic counseling for those with a familial cancer history (Maitra et al. 2006). The progression of PDAC is influenced by internal genetic changes and external inflammatory factors. Different mechanisms of controlled cell death, including apoptosis, necroptosis, ferroptosis, pyroptosis, and alkaliptosis, significantly influence PDAC biology and treatment responses. Therapeutically induced cell death can inhibit tumor growth and metastasis, but excessive damage may lead to an inflammatory, immunosuppressive environment that promotes tumor growth (Chen et al. 2021). PC increased the molecular mechanisms, uncovering genetic anomalies such as point mutations and chromosomal abnormalities that affect disease onset and progression (Hansel et al. 2003). The lysosome‐mediated degradation process known as autophagy is crucial for energy metabolism and cellular defense. PC show higher levels of autophagy. The interplay between autophagy and PC development highlights risk factors such as alcohol consumption, smoking, and pancreatitis, as well as the tumor microenvironment and molecular mechanisms involving K‐Ras and p53. The HMGB1‐RAGE pathway plays a significant role in regulating autophagy, with recent studies demonstrating that pharmacological inhibitors, such as chloroquine, and RNA interference targeting key autophagy genes can inhibit autophagy and impact chemotherapy sensitivity in PC (Kang and Tang 2012). A study identifies histological subgroups of pancreatic neoplasms that transition slowly from benign to malignant forms. It demonstrates various factors, including mutations (e.g., p16/cdkn2a, k‐ras), molecular actions in rare tumors, and key pathways such as the PI3K‐Akt‐mTOR and VEGF pathways (Matthaios et al. 2011). In this study, 94% of clinical pancreatic adenocarcinoma specimens showed significantly elevated ZIP4 mRNA levels compared with surrounding normal tissues, and ZIP4 mRNA expression was higher in cancer cells than in healthy ductal epithelium. This upregulation of ZIP4 may influence the pathophysiology of PC. ZIP4 overexpression increased tumor volume significantly in a nude mouse model, as well as intracellular zinc levels and cell proliferation. The orthotopic model showed increased tumor weight and ascites incidence associated with ZIP4 overexpression. The crucial role of abnormal ZIP4 expression in PC development suggests potential therapeutic targets to inhibit cancer progression (Li et al. 2007).

**FIGURE 1:**
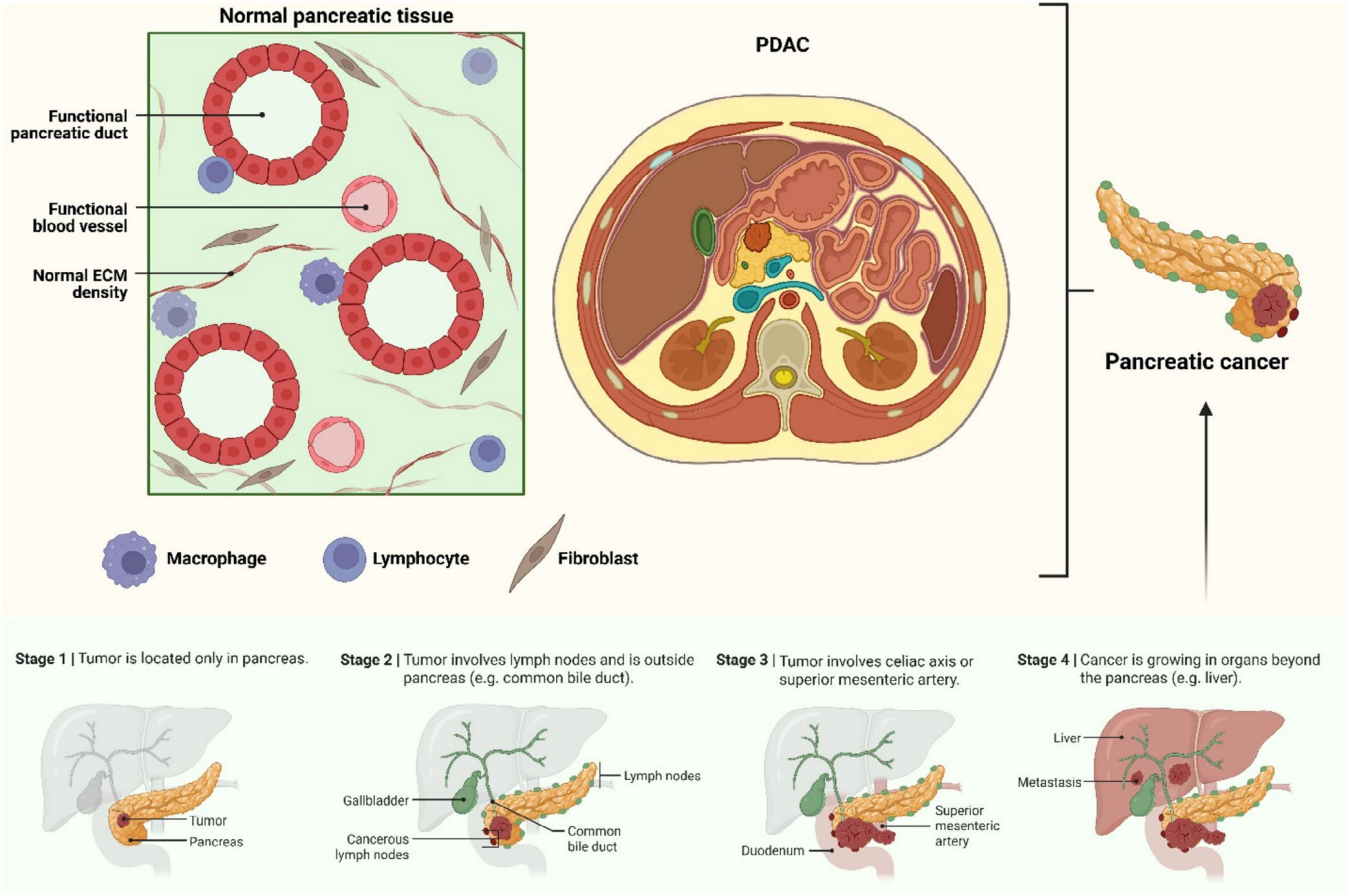
Structural and pathological progression of pancreatic ductal adenocarcinoma (PDAC). The figure illustrates the transition from normal pancreatic tissue to PDAC and its clinical stages. The comparison shows that the normal pancreas features a balanced extracellular matrix density and functional blood vessels, whereas PDAC is characterized by a dense stroma, obstructed ducts, and disorganized tumor structure, influenced by activated fibroblasts and immune cells. Stage I describes a pancreatic tumor localized to the pancreas. In Stage II, the tumor begins to invade adjacent structures, such as the common bile duct and lymph nodes. Stage III indicates involvement of significant blood vessels, such as the superior mesenteric artery or the celiac axis. Finally, Stage IV denotes advanced metastatic disease with spread to distant organs, particularly the liver.

## 4 Phytochemicals in PC

### 4.1 Curcumin

Curcumin has demonstrated anticancer benefits against PC by regulating multiple molecular targets. Preclinical studies reveal strong cytotoxic effects on PC cell lines. Additionally, in vivo studies show that curcumin's antiproliferative properties arise from its ability to stimulate apoptosis while suppressing OS and angiogenesis (Bimonte et al. 2016). Additionally, curcumin, known for its potent NF‐κB inhibitory activity, was evaluated for its effects on PC. Patients received 8 g of oral curcumin daily, with monitoring of NF‐κB expression, COX‐2 levels, and serum cytokines. Curcumin demonstrated limited oral bioavailability in 21 of the 25 enrolled patients, with circulating forms mostly consisting of glucuronidated and sulfated variants. Curcumin reduced NF‐κB and COX‐2 expression in peripheral blood mononuclear cells, and drug levels remained initially stable despite variability among patients (Dhillon et al. 2008). Curcumin enhances the anticancer effects of gemcitabine, as demonstrated in a study of 17 patients with PC. Patients received gemcitabine (1000 mg/m^2^ IV weekly for three out of 4 weeks) alongside curcumin (8000 mg orally daily). However, 29% of participants discontinued curcumin due to severe abdominal pain, prompting a dosage reduction for others. Among 11 evaluated patients, 36% maintained stable disease, while 55% experienced tumor progression, and 9% showed partial remission. Low adherence to high‐dose curcumin may limit its effectiveness when combined with gemcitabine (Epelbaum et al. 2010). Furthermore, curcumin alters microRNA (miRNA) expression in pancreatic cells. Specifically, it up‐regulates miRNA‐22 and down‐regulates miRNA‐199a. Inhibition of miRNA‐22 increases the expression of SP1 and ESR1, whereas miRNA‐22 overexpression reduces their levels in PxBC‐3 PC cells (Sun et al. 2008). A study developed a novel curcumin‐loaded magnetic NP (MNP‐curcumin) formulation to challenge significant issues in PC treatment. The MNP‐curcumin formulation was effectively internalized by human PC cells (HPAF‐II and Panc‐1) in a dose‐dependent manner, leading to suppression of colony formation and cell proliferation in colony formation and cell proliferation assays (Yallapu et al. 2013). In a study on PC, BxPC‐3 cells were treated with curcumin and docosahexaenoic acid (DHA), and the resulting xenografts were then formed in nude mice. These mice were given diets supplemented with curcumin or fish oil for 6 weeks. The combination of curcumin and DHA produced a synergistic effect, substantially increasing apoptosis and reducing cell growth. Mice on a diet of fish oil and curcumin exhibited significant tumor volume reductions of 25% and 43%, respectively, and over 72% when combined. The combined dietary intervention also led to increased p21 expression and decreased iNOS, COX‐2, and 5‐LOX levels. Curcumin and omega‐3 fatty acids may effectively suppress PC (Swamy et al. 2008). Curcumin and its analogues EF31 and UBS109 can disrupt angiogenesis in PC by affecting HIF‐1α and NF‐κB. Curcumin‐treated MIA PaCa‐2 or PANC‐1 cells inhibited HUVEC tube formation and angiogenesis in vivo. Treatment with EF31 and UBS109 inhibited the vascularization of Matrigel plugs in mice and significantly reduced levels of factors such as VEGF and angiopoietins in PC cell lines. Additionally, these treatments reduced transcription of HSP90, NF‐κB, and HIF‐1α, even in the presence of NF‐κB overexpression. Notably, EF31 and UBS109 led to the downregulation of HIF‐1α, Hsp90, COX‐2, and VEGF in tumor xenografts (Nagaraju et al. 2015). Moreover, curcumin exhibits multiple anticancer effects, including antiangiogenesis, induction of apoptosis, and growth suppression in cancer cells and tumors. In a study involving nude mice with L3.6pL xenografts, curcumin significantly reduced tumor formation and the growth of Panc28 and L3.6pL PC cells. The study explored the relationship between Sp and NF‐κB factors, both of which regulate genes associated with cancer, such as cyclin D1 and survivin. Additionally, curcumin altered mitochondrial membrane potential and generated ROS, further indicating that its anticancer effects depend on its mitochondriotoxic properties (Jutooru et al. 2010). Curcumin (Figure 2) has potential in promoting autophagy in patients with PC. Higher concentrations of curcumin are associated with increased autophagic cell death (Zhu and Bu 2017). A study investigates the effects of curcumin on TGF‐β1‐induced EMT in PC PANC‐1 cells. TGF‐β1 treatment increased vimentin, GLI1, and Shh levels and decreased E‐cadherin expression, indicative of EMT. Curcumin treatment significantly inhibited cell proliferation, induced apoptosis, and reduced the expression of EMT‐related markers. Curcumin can reverse EMT and may target the Shh‐GLI1 signaling pathway as a potential mechanism for its effects (Sun et al. 2013). Curcumin inhibits tumor growth and angiogenesis by modifying cell signaling pathways. In an investigation of its effects on MIA PaCa‐2 PC cells, curcumin was found to induce apoptosis and suppress cell growth in vitro (Bimonte et al. 2013). FLLL11 and FLLL12, curcumin analogues, were studied for their potency against human PC cell lines. Their IC_50_ values ranged from 0.28–3.2 μmol/L for FLLL11 and 0.91–3.43 μmol/L for FLLL12, demonstrating greater effectiveness than curcumin, which had IC_50_ values between 8.67 and 20.35 μmol/L. All three compounds reduced cell viability, but FLLL11 and FLLL12 also inhibited the activation of the AKT and STAT3 pathways and induced higher levels of apoptosis, as shown by increased PARP and caspase‐3 cleavage. FLLL11 and FLLL12 may be more effective chemopreventive or therapeutic agents for PC than curcumin (Friedman et al. 2009). Curcumin reduced cyclin D1 expression and cell proliferation, correlated with increased p21 and p27. Additionally, it enhanced caspase‐3/9 activation and lowered the Bcl‐2/Bax ratio, promoting apoptosis. It also elevates FOXO1 expression by inhibiting the PI3K/Akt pathway, leading to cell‐cycle arrest and apoptosis (Zhao et al. 2015). Furthermore, curcumin prevents EMT in PC by inhibiting CAFs, which promote cell migration and metastasis. It reverses EMT phenotypes in PC cells, reducing mesenchymal properties of CAFs and preventing cancer cell migration and metastasis (Q. Wang et al. 2017). Curcumin, a Jab1 inhibitor, reduces tumor growth. In PC cells, this PEGylated curcumin notably decreased cell proliferation, induced abnormal multinucleated cells, and led to increased mitotic phase arrest, indicating an impact on cell cycle progression. Additionally, PEGylated curcumin suppressed Jab1/CSN‐associated kinase activity, improving the stability of Jab1 target proteins. In cells with Jab1 knockdown, the growth inhibition effects of PEGylated curcumin were reduced. PEGylated curcumin inhibits Jab1/CSN activity to disrupt cell division, while also enhancing the effectiveness of gemcitabine against PC cells. Overall, the PEG‐conjugated curcumin exhibits greater efficacy in inhibiting PC cell growth compared to free curcumin (Li et al. 2009). Moreover, curcumin enhanced apoptosis in PC cells via the PARP/caspase‐3 pathway and amplified the pro‐apoptotic effects of both chemotherapy agents. Furthermore, it significantly reduced PC cell metastasis by upregulating TIMP1/TIMP2 and downregulating MMP2/MMP9/N‐cadherin (Liu, Ying, et al. 2020). Curcumin has shown antitumor effects in PC by regulating miR‐7. This effect is linked to increased miR‐7 production and reduced SET8 expression. Thus, targeting miR‐7 with curcumin may provide a new therapeutic approach for treating PC (Ma et al. 2014). Additionally, curcumin reduces the expression of EZH2 and PVT1 in gemcitabine‐resistant PDAC cell lines, downregulates self‐renewal‐associated genes, and inhibits spheroid formation. It effectively prevents the development of gemcitabine‐resistant tumors (Yoshida et al. 2017).

**FIGURE 2:**
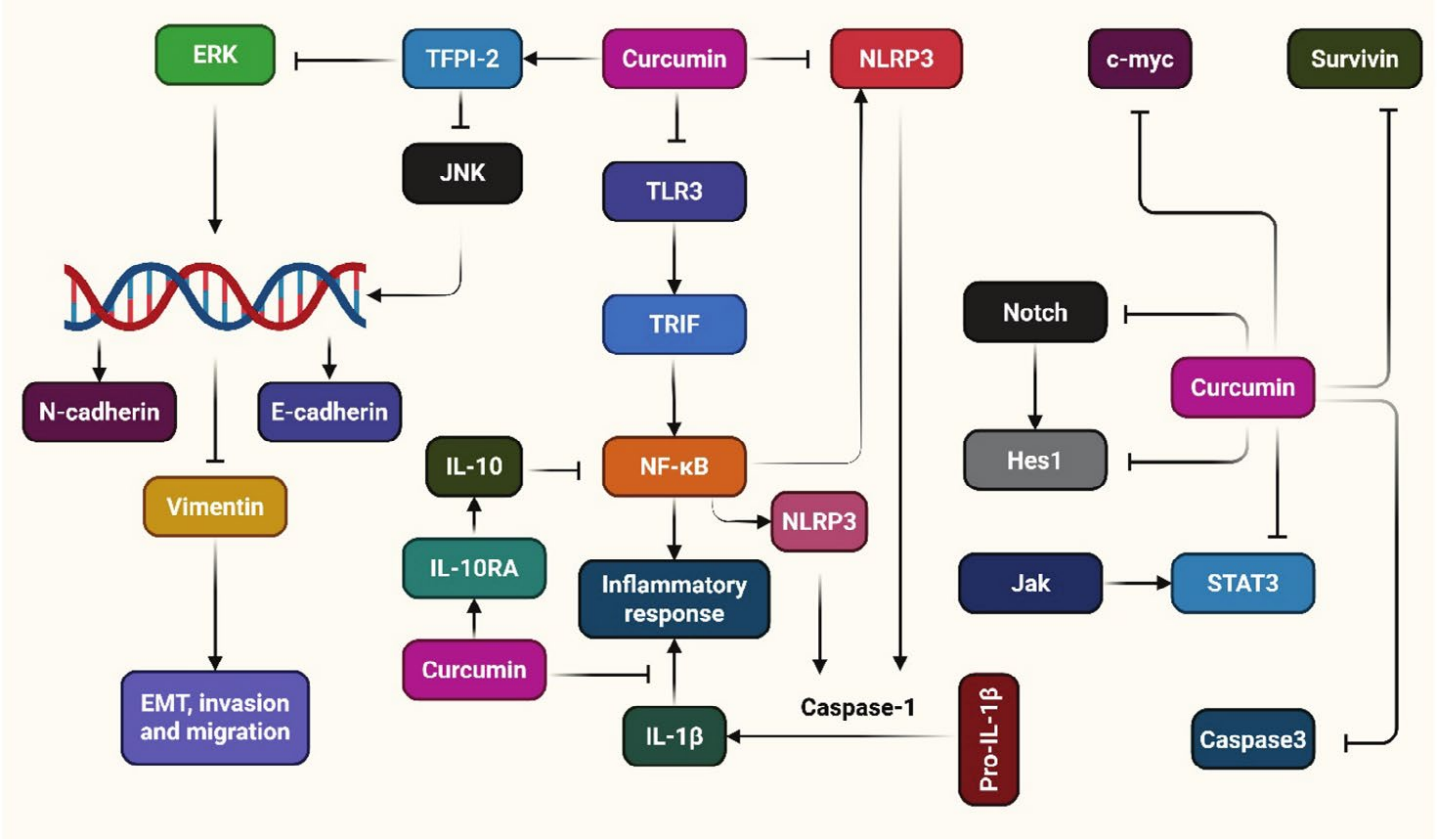
Multitarget molecular mechanisms of curcumin in PC signaling pathways. Curcumin exhibits multitarget regulatory effects on oncogenic and inflammatory signaling pathways involved in PC. It suppresses tumor cell invasion and migration by downregulating N‐cadherin and vimentin while upregulating E‐cadherin, modulating the ERK/TFPI‐2/JNK axis. Additionally, it reduces NF‐κB activation through the TLR3/TRIF/NF‐κB pathway and controls cytokine signaling by lowering pro‐inflammatory mediators such as IL‐1β and enhancing IL‐10 activity. Curcumin also inhibits the JAK/STAT3 survival pathway, reducing STAT3 activation and downstream targets such as survivin and c‐Myc, while interfering with Notch signaling to reduce tumor proliferation. Finally, it promotes apoptosis via caspase‐3 activation, highlighting its pleiotropic properties as a therapeutic agent for PC.

### 4.2 Quercetin

Quercetin suppresses self‐renewal in PC stem cell models. It reverses apoptosis resistance and decreases ALDH1 activity, with synergistic effects when combined with sulforaphane from broccoli. It also increased NF‐κB binding but inhibited EMT‐related proteins, further blocking early metastasis signaling. It inhibited cancer stem cell‐related xenograft growth, decreased angiogenesis, and induced apoptosis, with sulforaphane enhancing these effects without damaging normal cells (Zhou et al. 2010). Additionally, quercetin induced apoptosis and inhibited cancer cell growth, and oral supplementation reduced the growth of orthotopic pancreatic xenografts. Combining gemcitabine with quercetin showed no additional effects compared to quercetin alone. Oral quercetin treatment effectively prevented the formation of orthotopic PC in a nude mouse model (Angst et al. 2013). Quercetin is utilized as an adjuvant or alternative treatment for PC by influencing biological processes such as autophagy, apoptosis, and OS. Therapeutic effects through the modulation of molecular signaling pathways associated with cell growth, proliferation, antioxidative stress, EMT, apoptosis induction, and autophagy (Asgharian et al. 2021). Furthermore, quercetin modulates cell death and enhances gemcitabine efficacy by reducing RAGE levels, particularly in gemcitabine‐resistant PC cells. Specifically, in gemcitabine‐resistant cells, quercetin mimicked the effects of RAGE silencing by lowering RAGE expression, facilitating cell‐cycle arrest, autophagy, and apoptosis, and increasing gemcitabine sensitivity. Quercetin's ability to influence autophagy and apoptotic pathways by inhibiting RAGE expression in PC cells (Lan et al. 2019). The interactions of quercetin with PC stem‐like cells were investigated using a sphere‐formation culture system to compare these interactions with those of parent PC cells. Stem‐like cells exhibit heightened characteristics of cancer stem cells and increased gemcitabine resistance. Parent cells exhibited β‐catenin overexpression, whereas quercetin modulated its expression in stem‐like cells, thereby decreasing proliferation, invasion, and self‐renewal. Ultimately, targeting β‐catenin with quercetin may enhance treatment outcomes for PC patients (Cao et al. 2015). A synergistic effect was observed with gemcitabine and quercetin in inhibiting PC cell migration. Hyaluronic acid‐decorated NPs improved quercetin's anti‐inflammatory effects by reducing interleukin levels, which are crucial in the development, metastasis, and drug resistance of PC cells (Serri et al. 2019). Quercetin activated JNK, promoting cFLIP degradation via the proteasome pathway. It facilitates TRAIL‐induced apoptosis by inducing cFLIP turnover through JNK activation (Kim et al. 2016). A study investigated the apoptotic signaling pathway in human PC cells to evaluate quercetin's chemotherapeutic effects against cancer. Various anticancer drugs, including Adriamycin, cisplatin, and gemcitabine, were used. Quercetin (50 μg/mL for 24 h) induced apoptosis in PANC‐1 cells, leading to increased sub‐G0/G1 cell‐cycle phases and mitochondrial dysfunction, unlike the other tested drugs. Notably, quercetin increased intracellular Ca^2+^ levels and the expression of stress proteins, contributing to cytotoxic effects through ROS generation. Quercetin enhances the sensitivity of PC cells to chemotherapy (Lee et al. 2013). Quercetin has shown potential to inhibit tumor cell growth, but its effectiveness is limited by low bioavailability and poor solubility. HQL's ability to degrade HA in the tumor microenvironment facilitated NP penetration and contributed to its antitumor efficacy (Sun et al. 2025). Quercetin‐3‐*O*‐glucoside may enhance gemcitabine's chemotherapeutic effects and inhibit metastasis, determined by various growth factors in PC (Lee et al. 2016). By inhibiting EGFR signaling, quercetin 3‐O‐glucoside significantly reduced the migratory activity of human PC cells stimulated by EGF. Its antimigratory effects were evident at low doses and more noticeable than those of other quercetin forms. This compound exerted its antitumor effects through selective inhibition of key signaling pathways. Additionally, quercetin 3‐*O*‐glucoside, when combined with gemcitabine, produced a synergistic reduction in cell migration (Lee et al. 2015). Quercetin targets SHH signaling and shows potential as a therapeutic agent for PDA. The effects of quercetin were linked to reduced SHH activity, with Gli2 being essential for quercetin's action. Quercetin's benefits were reversed with increased SHH activity, which also promoted EMT through TGF‐β1/Smad2/3 signaling. Quercetin effectively induces apoptosis and reduces proliferation, migration, and invasion in PCCs by blocking SHH and TGF‐β/Smad pathways, indicating its viability as a treatment for PDA (Guo et al. 2021). Additionally, quercetin modulates lipid metabolism via the STAT3 pathway, thereby enhancing the therapeutic effects of gemcitabine (Huang et al. 2025). Quercetin induces stromal infiltration in PDAC by inhibiting CAF activation through tumor‐stroma interactions. Two prognostic models for quercetin in PDAC patients were identified, proposing the ITGB4 gene as a potential therapeutic target (Lei et al. 2024). Another study evaluated the pro‐apoptotic and antiproliferative effects of quercetin on human PC cells, both alone and in combination with daunorubicin, using EPP85‐181P (sensitive) and EPP85‐181RDB (resistant) cell lines. Quercetin exhibits concentration‐dependent cytotoxicity on both cell lines, potentially increasing the sensitivity of daunorubicin‐resistant EPP85‐181RDB cells. It overcomes chemotherapy resistance in neoplastic cells (Borska et al. 2010).

### 4.3 Resveratrol

Resveratrol demonstrates chemopreventive and therapeutic benefits in PC by suppressing cell growth, inducing apoptosis, inhibiting the cell cycle, and preventing metastasis and invasion. It also suppresses the proliferation of PC stem cells and enhances chemoradiosensitization (Xu et al. 2015). Additionally, resveratrol inhibits EMT in PC cells, which is crucial for tumor propagation. It targets the PI‐3K/Akt/NF‐κB signaling pathway to inhibit cancer progression, indicating its potential as an anticancer agent for PC (Li et al. 2013). In a study assessing resveratrol's effects on PC, two human PC cell lines, PANC‐1 and AsPC‐1, were treated with resveratrol. Results indicated a concentration‐ and time‐dependent reduction in cell growth, with significant decreases in cell numbers observed (Ding and Adrian 2002). Resveratrol directly binds to LTA4H, inhibiting LTB4 synthesis and LTB4 receptor (BLT1) signaling, thereby decreasing cancer cell proliferation. Resveratrol was found to be more effective than bestatin, an LTA4H inhibitor. Moreover, LTA4H knockdown reduced resveratrol's effectiveness. LTA4H is a significant target for resveratrol's anticancer effects (Oi et al. 2010). Furthermore, resveratrol exhibits potential anticancer properties against PC. It elevated Bax expression, induced apoptosis, inhibited tumor formation, and increased VEGF‐B levels. VEGF‐B siRNA intervention resulted in a higher apoptotic rate and reduced tumor activation via GSK‐3β. Notably, the resveratrol and VEGF‐B siRNA combination showed a significantly higher apoptotic rate than either treatment alone. Resveratrol demonstrates a dominant anticancer effect by upregulating Bax and also activating cancer processes via VEGF‐B (Yang et al. 2014). Resveratrol inhibited the activation of the NF‐κB signaling pathway in KC mouse pancreatic tissues, reducing the growth of precancerous lesions. It reduced ADM/PanIN development and the severity of cerulein‐induced pancreatitis, likely by inactivating NF‐κB. Furthermore, resveratrol's suppression of NF‐κB activity contributed to reduced PC tumorigenesis (Qian et al. 2020). Moreover, resveratrol inhibited cell viability in a dose‐dependent manner. Additionally, it reduced miR‐21 expression, thereby decreasing BCL‐2 protein levels. Its inhibition of miR‐21 influences BCL‐2 expression and apoptotic processes in these cancer cells (Liu, Liang, et al. 2013). Resveratrol overcomes gemcitabine‐induced resistance in PC. This study investigated SREBP1's role in enhancing stemness in gemcitabine‐treated cancer cells and the effects of resveratrol on chemosensitivity. Resveratrol reduced lipid production via SREBP1, increased gemcitabine sensitivity, and reduced the expression of cancer stem cell markers when SREBP1 was knocked down (Golkar et al. 2007). Additionally, resveratrol significantly affects the invasion and migration of PC cells, particularly Panc‐1 cells, under high‐glucose conditions. It reduces ROS and H_2_O_2_ levels and inhibits the proliferation, migration, and invasion of PC cells under hyperglycemic conditions (Cao et al. 2016). The hedgehog signaling pathway may mediate resveratrol's inhibitory effects on PC cell viability (Mo et al. 2012). Resveratrol impacts on PC cell invasion, hypoxia‐induced ROS formation, and Hh signaling activation. It reduced hypoxia‐induced expression of HIF‐1α and proteins associated with metastasis, such as MMP2 and uPA (Li et al. 2016). A study investigates the anticancer properties of triacetylresveratrol compared with resveratrol in PC cells. Notably, triacetylresveratrol and resveratrol also inhibited the nuclear translocation of these proteins and their interaction in PANC‐1 cells (Duan et al. 2016). Moreover, resveratrol inhibits NAF‐1 production in PC cells by activating Nrf2 signaling, thereby increasing cellular ROS levels (Cheng et al. 2018). Resveratrol analogue, demonstrating notable antiproliferative effects via DNA damage, apoptosis, and disruption of the cell cycle (Florio et al. 2023).

### 4.4 Epigallocatechin‐3‐Gallate

EGCG was evaluated for its therapeutic potential in PC, revealing its effects on the PI3K/Akt/mTOR signaling pathway in PTEN‐knockdown PC cells (Liu et al. 2016). Additionally, EGCG prevents the migration and invasion of PC cells. Furthermore, it enhanced the efficacy of gemcitabine by altering EMT markers. Overall, it also increases sensitivity to gemcitabine in combating PC by inhibiting cell migration and invasion (Wei et al. 2019). EGCG enhances cytochrome c release into the cytosol through Bax oligomerization and mitochondrial membrane depolarization. It may downregulate XIAP, facilitating downstream caspase activation. Additionally, it also increases ROS levels and activates JNK. Notably, antioxidants such as N‐acetylcysteine can prevent EGCG‐induced apoptosis by inhibiting the JNK pathway (Qanungo et al. 2005). Moreover, combining EGCG with TRAIL enhances procaspase‐3 cleavage and apoptosis, significantly reducing the proliferation of MIA PaCa‐2 cells (Basu and Haldar 2009). EGCG suppresses human PC cells and, when combined with bleomycin, induces apoptosis, exhibiting antitumor effects. The combination of EGCG and bleomycin significantly inhibits the proliferation of MIA PaCa‐2 cell lines in a dose‐dependent manner (Bimonte et al. 2015). Furthermore, EGCG prevents PC in vitro and, when combined with pterostilbene, shows additive effects on cell proliferation. EGCG and pterostilbene could enhance conventional PC treatments by differentially modulating apoptotic pathways (Kostin et al. 2012). In vitro experiments on MiaPaCa‐2 and PANC‐1 cells showed that EGCG decreased HIF‐1α stability, leading to lower levels of glucose transporter‐1 and glycolytic enzymes, ultimately inhibiting cell growth and glycolysis. Further RNA interference studies suggested that EGCG‐mediated suppression of HIF‐1α involved both the insulin receptor and IGF1R pathways. In vivo studies in athymic mice confirmed that EGCG reduced tumor development and HIF‐1α levels. It inhibits PC cell activity and influences HIF‐1α through both dependent and independent mechanisms involving the insulin receptor and IGF1R (Hu et al. 2023). EGCG regulates the production of Raf kinase inhibitor protein (RKIP) and metastatic activity in AsPC‐1 PC cells through epigenetic modifications. It upregulated RKIP by inhibiting histone deacetylase activity and increasing histone H3 expression, while also reducing Snail expression, NF‐κB translocation, MMP activity, and invasion. E‐cadherin expression was enhanced post‐EGCG treatment. RKIP induction correlated with decreased ERK phosphorylation, and U0126 treatment further increased RKIP levels and inhibited ERK phosphorylation. Overall, EGCG effectively mitigates invasive metastasis in AsPC‐1 cells by controlling the RKIP/ERK/NF‐κB axis and/or RKIP/NF‐κB/Snail interactions (Kim and Kim 2013). Moreover, EGCG impacts STAT3 signaling in PC cells, suggesting its therapeutic potential when used with gemcitabine or the JAK3 inhibitor CP690550. The combination of gemcitabine and CP690550 enhances EGCG's efficacy by targeting the STAT3 pathway (Tang et al. 2012).

### 4.5 Berberine

BBR selectively inhibits cancer cell growth, particularly more than normal cell growth, through cell cycle inhibition, ROS generation, apoptosis, autophagy, DNA interactions, and alterations in gene expression (Abrams et al. 2019). Additionally, BBR inhibits DNA synthesis and cell proliferation in PDAC cells, delaying cell cycle progression in the G1 phase. In vivo, it reduced MiaPaCa‐2 cell growth. It also affects specific pathways that can be reversed by reducing AMPK subunits at lower doses; however, at higher concentrations, it exhibits AMPK‐independent effects (Ming et al. 2014). BBR exhibits a specific anticancer effect on PDAC in vitro. It inhibits the proliferation and stemness of PC stem cells through mechanisms including suppression of the EMT. In orthotopic tumors, it also reduces tumor growth and Ki67 expression, indicating reduced cellular proliferation (Table 1) (M. Liu et al. 2022). An mRNA‐based gene expression profiling study showed that BBR had a selective inhibitory effect on PC cells compared with normal cells. It activates P53 signaling pathways, regulates cell cycle checkpoints, and influences the BRCA1‐mediated DNA damage response. This cytotoxicity may stem from BBR's ability to intercalate into DNA and induce DNA strand breaks by inhibiting topoisomerase (Pinto‐Garcia et al. 2010). BBR has therapeutic potential in PC and is linked to dysregulated glycolysis and mitochondrial damage in cancer cells. It inhibits PC cell survival and metastasis. It also disrupted energy metabolism and damaged mitochondria in PC cells, notably affecting citrate metabolism and transport, which inhibited fatty acid production by regulating ACLY, ACO1, and SLC25A1 (Liu, Luo, et al. 2020). Emodin and BBR show potential in cancer prevention by influencing the ECM in PC. This synergistic effect extends to alterations in key signaling proteins associated with EGFR/ERK1,2/AKT pathways, EMT, and apoptosis (Xu et al. 2024). BBR enhances PDAC's sensitivity to gemcitabine. Rap1/PI3K‐Akt signaling helps BBR reverse gemcitabine chemoresistance in PDAC (Okuno et al. 2022). In vitro experiments revealed that BBR administration led to cell cycle arrest, inhibited colony formation, and reduced cell proliferation. Additionally, in mouse models, BBR exhibited a significant tumor‐suppressive effect without causing renal or hepatic toxicity. It prevents PDAC by specifically targeting inflammation‐related genes (Ruan et al. 2024). Furthermore, BBR may enhance TP53 expression while inhibiting cell cycle progression in PDAC cells. The importance of identifying TP53 mutation types is demonstrated in cancer patients before administering TP53‐stabilizing medications (Abrams et al. 2022).

**TABLE 1:** Phytochemicals are used to prevent and treat pancreatic cancer.

Phytochemicals	Signaling pathways	Findings	References
Curcumin	NF‐κB	Induced apoptosis and inhibited the proliferation of MIA PaCa‐2 cells.	Bimonte et al. (2013)
	ATM/Chk1	Induced G2/M cell cycle arrest and apoptosis in PC cells, significantly influenced by ATM/Chk1 signaling.	Sahu et al. (2009)
	PI3K/Akt	Enhanced FOXO1 expression by inhibiting the phosphoinositide 3‐kinase/Akt signaling pathway, leading to cell cycle arrest and apoptosis.	Zhao et al. (2015)
Quercetin	RAGE/PI3K/AKT/mTOR	Inhibited RAGE expression, contributing to its molecular mechanisms that control apoptosis and autophagy pathways while enhancing GEM chemosensitivity in PC cells.	Lan et al. (2019)
	EGFR	Inhibited EGF receptor signaling, thereby reducing cell migration in several human PC cell lines stimulated by EGF.	Lee et al. (2015)
	SHH and TGF‐β/Smad	Induced apoptosis in PCCs and reduced their proliferation, migration, and invasion by inhibiting the TGF‐β/Smad and SHH signaling pathways.	Guo et al. (2021)
Resveratrol	PI‐3 K/Akt/NF‐κB	Inhibited the migration and invasion of PC by blocking the PI‐3K/Akt/NF‐κB signaling pathway.	Li et al. (2013)
	ERK and p38 MAPK	Inhibited the ERK and p38 MAPK signaling pathways, reducing PC progression associated with hyperglycemia‐induced ROS.	Cao et al. (2016)
	ROS/Nrf2	Inhibited NAF‐1 expression in PC cells by inducing ROS production and activating Nrf2 signaling.	Cheng et al. (2018)
Epigallocatechin‐3‐gallate	Akt	Prevented PC cells from migrating and invading by inhibiting their proliferation, both in vitro and in vivo.	Wei et al. (2019)
	PI3K/AKT/mTOR	Inhibited PANC‐1 cell growth and induced apoptosis, influencing the PI3K/AKT/mTOR signaling pathway by increasing PTEN expression.	Liu, Wang, et al. (2013)
	STAT3	Inhibited the growth, invasion, and migration of PC cells and promoted apoptosis by targeting the STAT3 signaling pathway.	Tang et al. (2012)
Berberine	EMT	Inhibits the proliferation and stemness of PC stem cells by suppressing the EMT signaling pathway, leading to reduced tumor growth and Ki67 expression in orthotopic PC.	Liu et al. (2022)
	LAMB3‐EGFR	Exerted anticancer effects in PDAC by interfering with LAMB3 and modifying the EGFR‐signaling pathway.	Xu et al. (2024)
	Rap1/PI3K‐Akt	Regulated the Rap1/PI3K‐Akt signaling pathway, which contributes to overcoming chemoresistance associated with gemcitabine in PDAC.	Okuno et al. (2022)
Genistein	STAT3	Exhibited anticancer effects in PC cells through ROS‐mediated mitochondrial apoptosis, cell cycle arrest, and regulation of STAT3.	Bi et al. (2018)
Apigenin	PI3K/Akt	Suppressed the growth of human PC cells, potentially through the down‐regulation of GLUT‐1 expression via the PI3K/Akt signaling pathway.	Golkar et al. (2006)
	IKK‐β‐mediated NF‐κB	Induced apoptosis in PC cells, reduced cell proliferation, and inhibited growth in human PC xenografts.	Wu et al. (2014)
Luteolin	STAT3‐DPYD	Prevented cell proliferation in PC cells and correlates with decreased levels of DPYD and pSTAT3.	Kato et al. (2021)

### 4.6 Genistein

Genistein inhibits tumor growth in PC. Genistein therapy led to upregulation of Fbw7, a target of miR‐223, and a significant reduction in miR‐223 expression. This downregulation resulted in increased apoptosis and decreased proliferation in PC cells. Thus, genistein may exert antitumor effects by inhibiting miR‐223 in PC cells (Ma et al. 2013). Additionally, genistein enhances miR‐34a levels, leading to Notch‐1 downregulation, induction of apoptosis, and inhibition of cell growth. It acts as a non‐toxic agent that activates miR‐34a to curb PC cell proliferation (Xia et al. 2012). Genistein prevents proliferation in human PC cell lines and enhances the effect of chemotherapy, specifically gemcitabine. It enhances chemosensitivity in PC by inhibiting gemcitabine‐induced NF‐κB activation (Banerjee et al. 2005). Furthermore, genistein regulates the expression of the miRNA miR‐27a in PC cells. Genistein and miR‐27a have a synergistic effect on cancer progression, making miR‐27a a potential target for PC treatment, as inhibiting it aids in tumor suppression by genistein (Xia et al. 2014). Genistein decreased cell viability in Mia‐PaCa2 and PANC‐1 cancer cells with IC_50_ values of 20 and 25 μM, respectively. Its effects were less potent against noncancerous H6C7 cells (IC_50_: 120 μM) (Bi et al. 2018). Another study found genistein in PC using an orthotopic nude mouse model and in vitro tests. Intraperitoneal genistein treatment significantly increased apoptosis, prevented metastasis, and improved survival in vivo. Genistein treatment in vitro induces apoptosis in PC cell lines, primarily through caspase‐3 activation (Büchler et al. 2003). Genistein increased autophagy markers, including Beclin‐1, and decreased Bcl2 levels, thereby enhancing cell death when combined with 5‐FU. This combination significantly reduced tumor volume compared to 5‐FU alone (Suzuki et al. 2014). Moreover, genistein promotes EMT by increasing vimentin levels and decreasing E‐cadherin levels. TGF‐β1 utilizes multiple signaling pathways, including the Smad4‐dependent and p38 MAPK pathways, to mediate EMT and may facilitate genistein's anticancer properties (Lei and Zhang 2012). Genistein inhibits cancer cell growth without affecting healthy cells, potentially by deactivating NF‐κB activity, which is linked to chemoresistance to some chemotherapy drugs. Genistein pretreatment may improve the anticancer effectiveness of docetaxel and cisplatin (Li et al. 2004). Additionally, genistein reduces miR‐223 expression in gemcitabine‐resistant PC cells. The combined effects of genistein and a miR‐223 inhibitor decreased cell invasion and motility while enhancing sensitivity to gemcitabine. Additionally, these reversed EMT characteristics in resistant cells (Ma et al. 2016). Genistein inhibits tyrosine kinases and HIF‐1, a key regulator of VEGF gene expression. In a study on hypoxic cell culture and PC models, genistein treatment led to dose‐dependent decreases in VEGF protein secretion and HIF‐1 activation, as well as lower VEGF mRNA levels in both normoxic and hypoxic states. Genistein inhibits tumor growth in xenograft tumors under hypoxic conditions (Büchler et al. 2004). Furthermore, genistein‐resistant Panc‐1 and PaCa PC cells exhibit low DEPTOR expression. Treatment with everolimus downregulates DEPTOR, thereby activating the PI3K/AKT/mTOR pathway and enhancing apoptosis, LDH release, and caspase‐3 activity. DEPTOR overexpression reduced the IC_50_ of genistein and enhanced cell death, indicating increased sensitivity when combined with everolimus in both in vitro and mouse models. ELK1 lowers DEPTOR levels, promoting mTOR phosphorylation and contributing to drug resistance in PC cells (Li et al. 2021). STAT3 is crucial for differentiation and proliferation, and its constitutive activation is found in PC. Tumor cell lines Panc‐1 and MIA PaCa‐2 also exhibit active STAT3, modulated by genistein and indole‐3‐carbinol (I3C) at concentrations above 10 μM, with I3C inducing apoptosis (Lian et al. 2004).

### 4.7 Apigenin

Apigenin (Figure 3) exhibits significant anticancer potential with anti‐inflammatory and antioxidant properties. Furthermore, it affects molecular pathways such as GLUT‐1, VEGF, and HIF, thereby enhancing the sensitivity of PC cells to chemotherapy (Ashrafizadeh et al. 2020). Additionally, apigenin was tested in a trial to evaluate its effectiveness in combination with gemcitabine for PC treatment. The combination therapy enhanced antitumor efficacy by inhibiting Akt activation and reducing NF‐κB activity in PC cell lines and tumor tissues, leading to increased growth inhibition and apoptosis both in vitro and in vivo (Lee et al. 2008). Apigenin demonstrated concentration‐ and time‐dependent inhibition of DNA synthesis and cell proliferation in four PC cell lines, causing G2/M phase cell cycle arrest. It reduced levels of key proteins involved in the G2/M transition, including phosphorylated CDC2, cyclin A, cyclin B, and CDC25. It also prevents proliferation through G2/M arrest (Ujiki et al. 2006). Furthermore, apigenin exhibits antiangiogenic and antiproliferative properties, influencing hypoxia‐responsive genes critical for PC cell growth (Melstrom et al. 2011). Apigenin stabilizes Ikaros expression in isolated splenocytes and enhances its levels in vivo. It decreased CK2 activity, restored various Ikaros isoforms, and increased CD4+ and CD8+ T cells while reducing Tregs. Splenocytes from TB‐apigenin mice enhanced allogeneic CD8+ T cell responses and increased IFN‐γ production. CK2 regulates Ikaros in PC, indicating the potential of apigenin as a treatment to maintain T cell homeostasis by preserving Ikaros expression and function (Nelson et al. 2017). Moreover, apigenin induces apoptosis and reduces cell proliferation by inhibiting IκB‐α phosphorylation, the translocation of p65 and p50, and downregulating NF‐κB DNA‐binding and transcriptional activity. Overexpression of IKK‐β reduced apigenin's effects, while IKK inhibition enhanced its anticancer properties. In xenograft models, apigenin (30 mg/kg) effectively inhibited PC growth and IKK‐β activation (Wu et al. 2014). Apigenin was investigated for its antiproliferative effects on human PC cells BxPC‐3 and PANC‐1, targeting the glycogen synthase kinase‐3β/NF‐κB signaling pathway. It altered the expression of apoptotic proteins and triggered mitochondrial apoptosis (Johnson and De Mejia 2013). A study investigated the molecular mechanisms underlying apigenin's cytotoxic effects on PC cells and its influence on NK cell proliferation. It enhances NK cell proliferation and cytotoxicity, likely by modulating the JNK and ERK signaling pathways (Feng et al. 2023). Apigenin inhibits PC cell growth by downregulating the protein geminin, which binds to and inhibits the replication factor Cdt1. Research using PC cell lines CD18 and S2013 indicated that apigenin reduces both mRNA and protein levels of geminin and Cdc6, and does not affect Cdt1 and MCM7 expressions. Additionally, apigenin‐mediated suppression of geminin promoter activity was confirmed in HeLa cells (Salabat et al. 2008). Additionally, apigenin inhibits glucose uptake and reduces GLUT‐1 expression in human PC cells (CD18 and S2‐013). Treatment with apigenin led to a dose‐dependent reduction in glucose uptake and in GLUT‐1 mRNA and protein levels, suggesting that the PI3K/Akt pathway mediates these effects (Melstrom et al. 2008). Furthermore, apigenin's inhibition led to the degradation of MMP‐9, CDK4, and Akt, resulting in increased intracellular ROS and inhibited cell migration and proliferation in PC cells (He et al. 2015). Another study investigated the effects of combining apigenin and gemcitabine on cell proliferation, cell cycle, and resistance in human PC cells. The combination therapy significantly reduced cell growth and enhanced apoptosis compared with individual treatments, resulting in S‐ and G2/M‐phase arrest. Additionally, apigenin inhibited gemcitabine‐induced pAkt expression and suppressed growth in gemcitabine‐resistant cells. The combination therapy may effectively combat PC by inducing apoptosis and cell cycle arrest (Strouch et al. 2009). Apigenin inhibits PC growth and influences p53 activation. It increased apoptosis (up to 100‐fold) in treated cells and enhanced p53 post‐translational modifications (King et al. 2010). The PI3K/Akt pathway is inhibited by apigenin, reducing GLUT‐1 expression and preventing the growth of PC cells. Apigenin and pAkt inhibitors (LY294022 and Wortmannin) both decreased GLUT‐1 expression (L Golkar et al. 2006). In this study, four PC cell lines (AsPC‐1, CD18, MiaPaCa2, and S2013) were treated with apigenin at concentrations of 6.25–100 μM, either alone or in combination with gemcitabine (100 μM). The combination of apigenin and gemcitabine induced 32.5% apoptosis (M. Ujiki et al. 2004).

**FIGURE 3:**
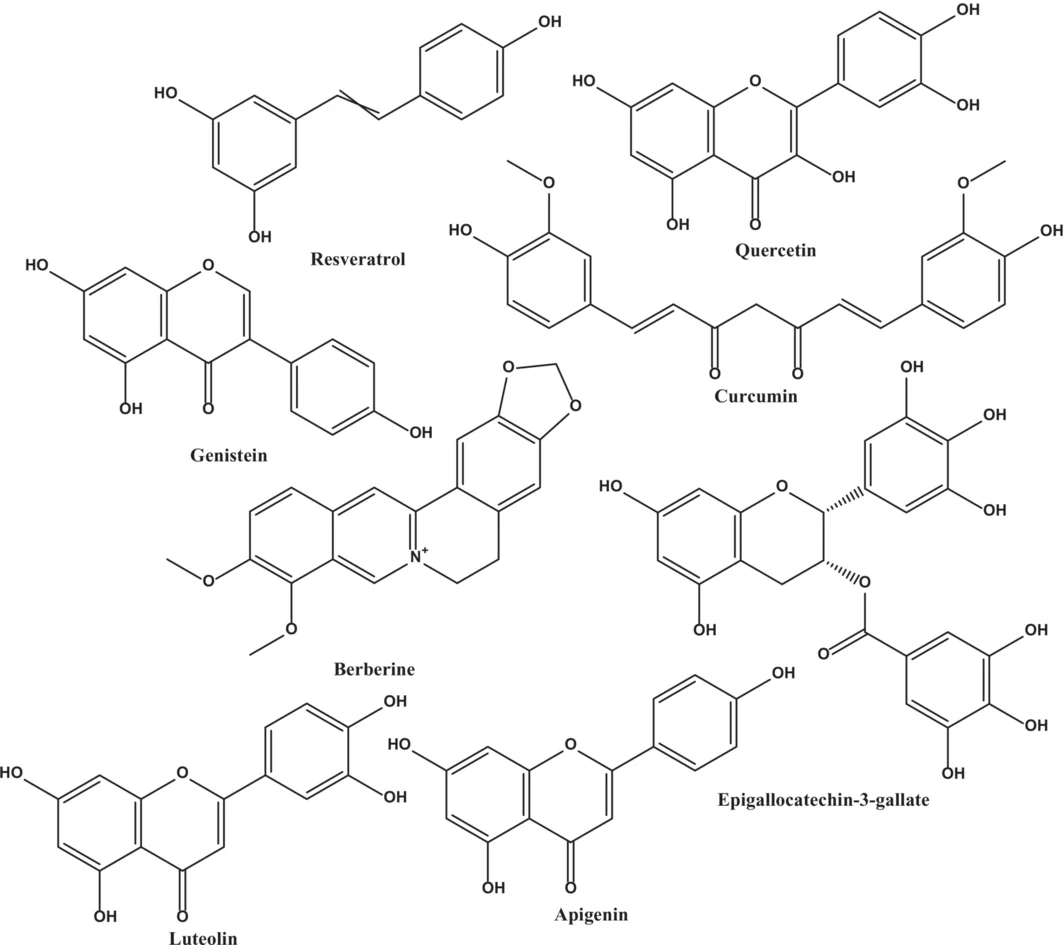
Chemical structure of phytochemicals which are responsible for preventing and treating PC.

### 4.8 Luteolin

Amplified BCL‐2 in SW1990 PC cells contributes to malignant cell survival and poor patient prognosis. Luteolin, a natural BCL‐2 inhibitor identified through structure‐based virtual ligand screening, shows dose‐dependent pro‐apoptotic effects in vitro. It promotes apoptosis in SW1990 cells by displacing BAX from BCL‐2, leading to mitochondrial permeabilization. In xenograft models, it also significantly reduced tumor growth (Li et al. 2018). A study investigated the effects of gemcitabine, luteolin, and their combination on PC growth in vivo. The combination of luteolin + gemcitabine significantly reduced tumor mass and decreased nuclear antigen expression in proliferating cells (Johnson et al. 2015). Luteolin and hesperetin were evaluated for their chemopreventive effects on pancreatic carcinogenesis using a BOP‐induced hamster PDAC model. Luteolin significantly reduced the incidence and growth of pancreatic intraepithelial neoplasia and inhibited the proliferation of both human and hamster PC cells in vitro. Its exposure correlated with reduced levels of DPYD and pSTAT3. DPYD levels in human PDAC cells influenced cell proliferation and pSTAT3 expression. Notably, high DPYD levels were associated with poor prognosis in human PDAC cases (Kato et al. 2021). In this study, the anticancer effects of luteolin on PC cells were investigated, with a focus on its ability to induce apoptosis. Three human PC cell lines (PANC‐1, CoLo‐357, BxPC‐3) were used, revealing characteristic apoptotic features following 24‐h luteolin treatment (Cai et al. 2012). A study developed an effervescent‐based nanocarrier to enhance the oral bioavailability of luteolin, a hydrophobic medication used in cancer treatment. The nanocarrier transforms into nanomicelles, thereby improving cellular uptake and permeability in vitro in Caco‐2 cells. The nanomicelles effectively downregulated VEGF‐A, FAK, TNF‐α, and Ki‐67 while activating caspase‐3, demonstrating strong anti‐PC effects compared to traditional drug delivery methods (Karole et al. 2024). Luteolin reduces DPYD expression in PDACs, inhibiting tumor development and affecting the breakdown of pyrimidines such as 5‐FU. PDAC cells with DPYD overexpression exhibit increased invasiveness and proliferation. Elevated levels of MMP9 and MEP1A, linked to metallopeptidase activity, were observed in these tumors. The combination of 5‐FU and luteolin has shown significant tumor‐suppressive effects in xenograft and KPPC mouse models (Kato et al. 2024).

## 5 Clinical Evidence

The therapeutic effects of curcumin treatment in patients with proven PC were assessed in a Phase II experiment. Twenty‐one out of 25 enrolled patients were evaluated for their response to 1 g caplets of curcumin. Patients took 8 g of oral curcumin daily until the condition progressed. The disease was reassessed every 2 months. The plasma curcumin levels remained mostly constant, ranging from 22 to 41 ng/mL. Clinical biological activities were observed in two patients: one sustained stable disease for over 18 months, while the other had a severe tumor regression lasting 1 month (Dhillon et al. 2008). In a clinical trial involving 17 patients with advanced PC, gemcitabine was administered alongside oral curcumin (8 g daily) for 3 weeks. Five patients (29%) discontinued due to severe abdominal pain, while two reduced their dosage. Among the 11 patients assessed, six experienced tumor progression, four had stable disease, and one achieved a partial response. Overall survival varied from 1 to 24 months (median 5 months), and time to tumor progression ranged from 1 to 12 months (median 2.5 months) (Epelbaum et al. 2010). In a study of 66 patients with advanced PC, those treated with curcumin (22 patients) experienced greater loss of muscle and subcutaneous fat compared to those who did not receive treatment (44 patients). Median survival for sarcopenic patients on curcumin was 169 days (range, 115–223), whereas untreated sarcopenic patients had a median survival of 299 days (range, 229–369), with a significant difference. No survival difference was found in non‐sarcopenic patients (Parsons et al. 2016). Rats administered oral curcumin at a dosage of 2 g/kg exhibited absorption and elimination half‐lives of 0.31 ± 0.07 and 1.00 ± 0.5 h, respectively. Human patients administered a 2 g dosage of curcumin showed undetectable serum curcumin levels (Shoba et al. 1998). Another Phase I/II clinical trial tested curcumin for patients with PC resistant to gemcitabine. Twenty‐one individuals were involved, and 8 g of curcumin were deemed suitable as it did not cause toxicity when combined with gemcitabine. Plasma curcumin levels in five evaluated patients ranged from 29 to 412 ng/mL. The one‐year survival rate was 19%, with a median survival duration of approximately 5.4 months (Kanai et al. 2011). In cancer clinical studies, theracurmin has shown potential as an effective treatment. A phase I study reported curcumin peak plasma levels between 324 and 440 ng/mL in 14 PC patients receiving Theracurmin, which includes 200 or 400 mg of curcumin alongside Gemcitabine (Kanai et al. 2013).

## 6 Conclusion and Future Perspectives

Phytochemicals exhibit significant anticancer effects through mechanisms such as inducing apoptosis, suppressing proliferation, inhibiting angiogenesis, and reversing EMT, by modifying several signaling pathways, including KRAS/MAPK, PI3K/Akt, NF‐κB, STAT3, Wnt/β‐catenin, and Hedgehog. Multitarget chemotherapeutics provide significant advantages over single‐target treatments by simultaneously targeting several oncogenic networks, which is, particularly, beneficial in cancers with poor outcomes and high resistance. Phytochemicals can work alongside conventional chemotherapeutic medicines, suggesting their potential as adjuvant treatments. Numerous phytochemicals face challenges such as limited tissue penetration, rapid metabolism, low bioavailability, and poor solubility. The development of standardized clinical guidelines is complicated by variations in purity, dose, and pharmacokinetics among trials. The development of advanced drug‐delivery methods, such as polymeric carriers, liposomes, and NPs, is crucial for enhancing the stability and targeted delivery of phytochemicals to PC. The effectiveness of treatment can be enhanced by incorporating phytochemicals into personalized medicine approaches, particularly for patients with advanced or chemoresistant diseases. Large, well‐planned clinical trials are essential for developing evidence‐based treatment guidelines and evaluating the long‐term benefits of combining phytochemicals with standard‐of‐care medicines. Phytochemicals show potential as multitarget drugs that may transform PC treatment. The review highlights key limitations affecting therapeutic efficacy, including rapid metabolism, poor bioavailability, and suboptimal pharmacokinetic profiles. Major obstacles to clinical translation include a lack of large‐scale, well‐designed clinical trials, diversity of study designs, and issues related to drug delivery and tumor heterogeneity. Furthermore, future prospects in therapeutic applicability emphasize the necessity for enhanced formulations, biomarker‐guided approaches, and strong clinical validation.

## Author Contributions

**Abdur Rauf:** conceptualization, supervision, data curation, methodology, investigation, writing – review and editing. **Abdullah S. M. Aljohani:** investigation, methodology, writing – original draft, data curation. **F. M. Shourav:** investigation, methodology, writing – original draft, data curation. **Waleed Al Abdulmonem:** investigation, methodology, data curation, writing – original draft. **Hanan A. Ogaly:** investigation, writing – original draft, methodology, data curation. **Kamala Badalova:** investigation, methodology, writing – original draft, data curation. **Marcello Iriti:** conceptualization, investigation, methodology, writing – review and editing, data curation, supervision. **Md. Rezaul Islam:** conceptualization, methodology, investigation, writing – original draft, data curation.

## Funding

This work was supported by King Khalid University (RGP2/82/47).

## Conflicts of Interest

The authors declare no conflicts of interest.

## Data Availability

The data that support the findings of this study are available from the corresponding author upon reasonable request.
